# Estimating the Benefits of Oral Cancer Screening: Challenges and Opportunities

**DOI:** 10.3390/cancers16234110

**Published:** 2024-12-08

**Authors:** Francesca Farina, Nicola Cirillo

**Affiliations:** 1 Melbourne Dental School, Faculty of Medicine, Dentistry and Health Sciences, The University of Melbourne, Carlton, VIC 3010, Australia; 2 STEMM Research, Liverpool L3 8HY, UK; 3 School of Dentistry, University of Jordan, Amman 11733, Jordan

**Keywords:** Oral cancer, early detection, screening, lead time, mortality, teledentistry, education

## Abstract

Screening is widely believed to facilitate early detection and improve health outcomes; however, recent evidence questions its overall benefits, highlighting the need for a critical assessment of cancer screening programs. The simplicity of clinical oral examination and the easy accessibility of the oral cavity make oral cancer a suitable candidate for opportunistic screening. Despite this uniqueness, studies that solidly assess the efficacy and cost-effectiveness of oral cancer screening are scanty, and the only large randomized trial undertaken so far shows limited evidence of mortality reduction through population screening. Research indicates that while early detection rates following screening may increase, they do not necessarily translate into decreased oral cancer deaths. This article emphasizes the need for a nuanced understanding of the benefits of screening, awareness of deceptive metrics and inherent biases in the context of early detection, and the importance of improving dentist education and introducing new technologies to enhance clinical decision-making. The review calls for a balanced and evidence-based approach to oral cancer screening, integrating it with broader oral health initiatives to maximize public health outcomes.

## Simple Summary

This article discusses the potential benefits and limitations of oral cancer screening. While early detection can be beneficial, there is limited evidence that screening programs significantly reduce deaths from oral cancer. The issue lies partly in how the effectiveness of screening programs is measured, with studies showing that higher detection rates do not necessarily translate to fewer deaths. The review highlights the importance of critical evaluation of screening programs and the need for improved dentist education and technology to reduce the burden of oral cancer. The authors advocate for a balanced, evidence-based approach to integrate screening into broader oral health strategies.

## 1. Introduction

The goal of early cancer diagnosis is to reduce morbidity and mortality for the disease, and screening has long been considered a vital strategy in this direction [1]. A cornerstone of preventive medicine and an example of secondary prevention, cancer screening offers the promise of early detection and improved survival for various malignancies. The central premise is that identifying cancer in its nascent stages, before symptoms appear, can lead to more effective treatment and, consequently, better patient outcomes [2]. This belief underpins numerous screening programs for cancers such as cervical, breast, and prostate [3]. However, the efficacy of population screening has been questioned, particularly in light of evidence suggesting that the benefits of screening may not be as significant as once thought [4].

Oral cancer screening presents a unique case, given its relative simplicity and the high prevalence of dental issues that facilitate opportunistic assessment—or screening [5]—of the oral mucosa. Despite these potential advantages, evidence supporting the efficacy of oral cancer screening in reducing mortality is limited [6]. Crucially, the literature pertaining to screening programs is fraught with methodological challenges that can distort their perceived effectiveness. For example, biases such as lead time and length bias can artificially inflate “success” metrics without genuinely improving patient outcomes. Studies from various regions, including India, Taiwan, and Cuba, suggest that while screening may increase the detection of early-stage cancers, it does not necessarily lead to a significant reduction in cancer deaths. This raises the question as to whether early detection and screening genuinely contribute to saving lives, as is often suggested.

In this review, we will delve into the complexities and challenges of cancer screening, with a particular focus on oral cancer. We will explore the biases that affect screening outcomes, evaluate the effectiveness of existing screening programs, and discuss the critical role general dentists should play in oral cancer detection. Our aim is to provide a balanced, non-ideological view of the current evidence and to propose potential strategies for improving cancer screening efficacy.

## 2. Assessing Whether Cancer Screening ”Saves Lives”

A screening test is a type of medical examination or procedure carried out on people in a specific, symptom-free group to determine their likelihood of having a certain disease [7]. In other words, screening is conducted on individuals who appear healthy, or at least without signs or symptoms of the illness being screened for. Modern medicine regards screening as the most important approach to diagnose certain diseases early and hence improve outcomes. For example, the management of advanced oral cavity cancer is complex and requires a multidisciplinary approach, often involving surgery and radiotherapy. In contrast, early-stage oral cancers can frequently be treated effectively with less invasive surgical procedures, resulting in lower costs, reduced side effects, and improved chances of a cure. However, among currently available screening tests for diseases where death is a common outcome, evidence shows that reductions in disease-specific mortality are uncommon and reductions in all-cause mortality are very rare or non-existent [8].

From a cancer policy perspective, a major objective of most screening tests is to reduce morbidity or mortality in the population group being screened. The NCI Dictionary of Cancer Terms suggests that “since screening may find diseases at an early stage, there may be a better chance of curing the disease” [9]. Although common wisdom aligns with the dictionary definition, this statement contains several assumptions. First, it assumes that screening indeed detects clinically meaningful diseases at an earlier stage; second, that there is an effective treatment available for the disease; and third, it presumes that patient outcomes improve when the disease is diagnosed earlier (for example, in terms of prognosis or quality of life). Meeting these assumptions is essential for early detection to have a positive impact on health outcomes [10]. Examples of cancer screening tests include the Pap smear for detecting cervical cancer, mammography for breast cancer detection, and the PSA test for identifying prostate cancer. While screening is regarded as the main reason for the observed decline in deaths from the aforementioned cancers in the last decades, very few of these screening tests have shown solid evidence of saving lives. In fact, there is a growing acknowledgement that the benefits of cancer screening in terms of lives saved are overstated, while the harms, such as overdiagnosis, overtreatment, as well as psychological and economic burden are downplayed or ignored in mainstream discussions [11]. This view is backed by the results of a most recent meta-analysis that estimated the extent of lifetime gained with cancer screening tests [12]. Somewhat surprisingly, the authors found that current evidence does not substantiate the claim that screening tests for common cancers save lives by extending lifetime, except possibly for colorectal cancer screening with sigmoidoscopy [12]. In particular, the meta-analysis revealed no significant lifespan extension from mammography—averaging literally zero days gained—even though this test is often considered the epitome of successful cancer screening. In another example, a recent meta-analysis investigated the efficacy and safety of prostate-specific antigen (PSA) testing to screen for prostate cancer and showed that it does not affect overall (all-cause) mortality [13].

But what is the right metric to be used when assessing the impact of population screening on mortality? There is ongoing debate about whether all-cause mortality, rather than cancer-specific mortality, should serve as the primary success measure in cancer screening. Advocates for using all-cause mortality argue that it provides a more complete assessment of a screening program’s impact, considering whether early detection genuinely extends life. This approach also takes into account the potential adverse effects of treatments on overall health. Measured by all-cause mortality or life-years gained, very few screening programs would be deemed beneficial. On the other hand, critics question the relevance of all-cause mortality as a success metric in cancer screening. A key argument against it is that many cancers have relatively low incidence, so changes in cancer-specific outcomes may have little effect on overall mortality rates at the population level. Furthermore, focusing on deaths from all causes may obscure the benefits of early detection for specific cancers. This is because the significant impact of preventing cancer-specific deaths in some individuals becomes diluted when averaged across the entire screened population, making the benefit appear smaller.

In summary, the belief that screening allows for the early detection of cancers, and hence saves lives, is deeply ingrained in both society and medical literature, despite it offering uncertain benefits in many cases. This is partly due to the use of biased and deceptive measures in reporting the effects of cancer screening.

## 3. Challenges with Estimating the Benefits of (Oral) Cancer Screening

The ultimate goal in detecting cancer, whether early or at a later stage, is to prevent or delay death from the disease, ideally without compromising quality of life. We do not diagnose cancer merely for the sake of diagnosis; our aim is to improve patient health outcomes. In this regard, the case of oral cancer is unique because, unlike many other conditions, both the disease and its interventions cannot be hidden, impacting not only mortality but also quality of life, social interactions, and psychological well-being, which must be taken into account when evaluating health outcomes. However, the most important, feared, and undesirable health outcome for a life-threatening condition like cancer remains death. This is why mortality data are crucial in shaping the decisions of individuals and healthcare providers. Therefore, the primary outcome one should strive for (and evaluate) is whether cancer screening and early diagnosis reduce deaths, extend people’s lives, or prevent the reduction of life quality due to the disease compared to the non-screened individuals. Unfortunately, this perspective is not widely held.

At a recent launch of the IARC Handbook of Oral Cancer Prevention [14,15], a panelist highlighted “an improvement in 5-year survival rates through early detection” as a success story of the program [15]. Sadly, increasing survival rates does not automatically translate into a reduction in cancer mortality. As it is known that early detection artificially inflates survival without impacting the actual number of deaths due to the disease [16], it is crucial to distinguish between improved survival and genuine reductions in mortality. The following paragraphs aim to explore these concepts in greater detail.

### 3.1. Lead Time Bias

**Figure 1:**
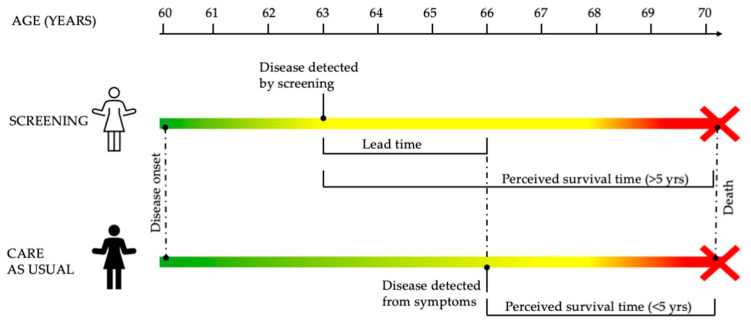
Schematic explanation of the lead time. In this hypothetical scenario, two individuals develop cancer at the same age (60 years). One undergoes screening, and the cancer is diagnosed when she is 63. This patient undergoes cancer treatment but still dies at 70, either due to cancer (e.g., recurrence or metastasis—disease-specific mortality) or other causes (all-cause mortality). The perceived survival time is 7 years (hence, she will be considered a cancer survivor according to the 5-year survival statistics). The other individual does not undergo screening, and the cancer is diagnosed when symptoms arise, at age 66. This patient dies at 70, with a perceived survival time of 4 years (according to survival statistics, this patient does not surpass the 5-year mark). However, both patients have died at the same age. The time elapsed between the early diagnosis of the symptomless cancer and the age at standard diagnosis (when symptoms are present) is the lead time. In this illustration, the green color of the disease timeline expresses that the patient who has not undergone screening has also lived longer without feeling “ill”.

Lead time is the period between the early detection of a disease, such as cancer, through screening and its usual clinical diagnosis. Lead time bias occurs when screening identifies cancer earlier than it would have been detected based on symptoms, leading to an apparent but misleading increase in survival time without necessarily extending the overall lifespan. For instance, if oral cancer is detected three years earlier through screening—perhaps due to the presence of an OPMD which demands strict follow ups—and the patient dies at the same age they would have without early detection, the survival time post-diagnosis artificially appears longer by three years (Figure 1). This is problematic because many outcomes measuring the effectiveness of screening rely on 3- or 5-year survival data, which can be inherently flawed due to lead time. To mitigate this bias, researchers often use cancer-specific and all-cause death rates in screened versus unscreened populations instead of just survival time after diagnosis.

In randomized controlled trials (RCTs), the start time is sometimes taken from the point of randomization rather than diagnosis [17]. Another method defined as “clinical detection survival analysis” measures survival time from when a patient first notices symptoms. However, this is not always feasible, especially in cases where symptomless early cancers are surgically removed. When eliminating lead time bias is not possible, statistical adjustments are sometimes made, such as using models to estimate lead time and adjust survival times accordingly [18]. Unfortunately, these corrections have not been applied in survival studies for oral cancer screening.

### 3.2. Length Bias

Length bias arises because screening is more likely to detect slower-growing, less aggressive cancers. These cancers have a longer pre-clinical phase (the period when the cancer exists but is asymptomatic and undetectable without screening), which makes them more likely to be detected in a screening program. This bias can lead to an overestimation of the benefits of screening, as the cancers most likely to be detected are also the ones with a better prognosis regardless of screening. In other words, it may appear that screened individuals do better and live longer with cancer, but this could be because they have less aggressive forms of the disease [19]. This bias would typically apply if oral cancers preceded by OPMDs were biologically less aggressive. To mitigate length bias, studies often compare the stage distribution of cancers detected by screening with those detected due to symptoms. They also compare the overall mortality (not just cancer-specific mortality) between screened and unscreened groups and take into account their growth patterns [20]. This comparison helps in understanding whether screening leads to a real reduction in death rates.

### 3.3. Other Key Biases and Challenges

There are of course other very well documented methodological challenges that make the design and interpretation of screening difficult. Briefly, these include: selection bias, which occurs when the group of individuals who choose to participate in screening is not representative of the general population (typically, individuals who opt for screening might be more health-conscious and have healthier lifestyles, potentially skewing the results of the screening program [21]); “sticky-diagnosis” and “slippery-linkage” biases, which are related to the accuracy and reliability of medical diagnoses and data linkage in health research and affect disease-specific mortality but not all-cause mortality [22]; false positives and false negatives occurring when a screening test incorrectly indicates the presence of cancer when it is not there (leading to unnecessary stress, anxiety, overtreatment, and healthcare costs) [23], or fails to detect a cancer that is present [24]; overdiagnosis bias, which occurs when screening correctly detects diseases or abnormalities, but these would not have caused symptoms or harm during a person’s lifetime; and population heterogeneity, which can affect the generalizability of screening results and the balance of risks and benefits [25].

Even when these factors are weighed and screening demonstrates a clear benefit for a certain health outcome, cost is a barrier to implementation, as screening programs require significant resources and funding. Therefore, evaluating the cost-effectiveness of these programs is crucial, especially in settings with limited healthcare resources. When a program is finally implemented, the effectiveness of screening is also dependent on the compliance of the target population: crucially, poor follow-up on abnormal screening results can undermine the potential benefits of early detection [26].

## 4. Efficacy and Cost-Effectiveness of Oral Cancer Screening

The physical examination of the head and neck in the screening for oral cancer is relatively simple and relies primarily on visual inspection. While the methodology is often ill described and imprecise, a recent systematic review [27] reported that most screening programs for oral cancer included visual inspection and palpation of the lips, oral cavity, and the most visible oropharyngeal sites. As visual screening may be easily conducted in remote and underserved areas, this scenario presents a vast opportunity for opportunistic screening and could help reduce health inequalities. The author (N.C) was involved in one such screening in 2008 in Kerala, India. The population being screened was composed of residents of villages that had served as controls in the famous Kerala Oral Cancer Screening Trial [28]. As this trial demonstrated that oral cancer deaths could be averted through screening in high-risk populations, the control group was also subjected to screening after the study was completed (Gigi Thomas, personal communication). There are unfortunately very few examples of randomized trials of oral cancer screening to date, and most available evidence is gathered from observational studies.

Another controversial aspect of estimating the benefits of oral cancer screening is whether the observed changes in cancer incidence are a result of screening programs. For example, in high-income countries where lip and oral cavity cancer mortality has significantly decreased over the last thirty years, there has been a concomitant decrease in incidence, suggesting a genuine reduction in oral cancer cases, possibly due to increased awareness of risk factors [29]. In these countries, no formal screening programs have been implemented. However, in countries with a low or middle socio-demographic index (SDI), there has been a substantial increase in incidence, which has been mirrored by a comparable rise in mortality, according to the Global Burden of Disease study, despite attempts to implement oral cancer and OPMD screening. Furthermore, since only a fraction of oral cancers are preceded by an observable OPMD, and the public health impact of early diagnosis prompted by OPMD on survival is limited [29], here we have not considered studies focused on OPMD screening.

### 4.1. Evidence from Randomized Controlled Trials

A large cluster-randomized controlled trial was conducted over 15 years in Kerala, South India, and involved four rounds of screening completed in 1998, 2002, 2004, and 2009 [28,30]. The program included 13 municipalities, divided into seven screened clusters (*n* = 96,517) and six control clusters (*n* = 95,356), and remains the most influential study in oral cancer screening. Conventional oral examination was used to detect potentially malignant and malignant lesions. Importantly, the primary outcome measure was mortality from mouth cancer in both the test and control cohorts.

The Kerala study found no significant difference in oral cancer mortality between the screened group (15.4/100,000 person-years) and the control group (17.1/100,000 person-years), with a relative risk (RR) of 0.88 (95% CI 0.69–1.12). However, over the 15-year period, there was a statistically significant 24% reduction in mortality among high-risk individuals (those using tobacco and/or alcohol), with the screened group showing a mortality rate of 30/100,000 person-years compared to 39/100,000 in the control group [30]. This unprecedented screening initiative saved 269 life years per 100,000 individuals overall and 1438 life years among the high-risk groups.

Even though some methodological issues have hindered the applicability of the results of this trial [31], to date, this is the only randomized controlled trial that assessed the benefits of oral cancer screening by evaluating a reduction in cancer mortality, as opposed to many other screening programs. This is somewhat surprising if we consider the easiness of an oral examination compared to undertaking mammography or CT scans for breast and lung cancer screening, respectively.

### 4.2. Evidence from Population Studies

Currently, most data available for screening derive from observational and population studies analyzing health repositories. One such study evaluated the outcomes of the Cuban oral cancer screening program [32]. Initiated in 1984, this was the first program of its kind, and required all subjects to undergo an annual oral examination by dentists. Between 1984 and 1990, this program identified 16% of the new oral cancer cases among the 4412 incident oral cancers recorded in Cuba during the time period. While the proportion of stage I cases at diagnosis increased, there was no significant change in overall oral cancer incidence and mortality attributable to the screening program during this time [32]. A subsequent case-control study conducted to evaluate the efficacy of this program revealed limited evidence towards a shift from advanced to early stages after the introduction of oral cancer screening in Cuba [33].

The authors of a population-based oral cancer screening program from Taiwan targeting high-risk groups reported a substantial reduction in risk of death from oral cancer in the screening group compared to no screening (RR = 0.53, which increases to 0.74 after adjusting for self-selection bias) [34]. These figures, however, may be misleading due to a number of methodological limitations. Briefly, between 2004 and 2009, a total of 4,234,393 adults with habits of cigarette smoking, betel quid chewing, or both, were targeted for oral cancer screening. A comparison was made between individuals who attended screening at least twice (*n* = 599,103) and non-attenders (*n* = 1,900,094). The authors reported that the overall incidence rate of oral cancer in the screened group (133.4 per 100,000) was statistically significantly lower than that in the non-screened group (190.9 per 100,000), which suggests that there are substantial baseline differences in the two groups; an alternative explanation would be that potentially malignant lesions were detected and removed in the screened group, but there is no indication that this was the case. The results from multivariate survival analysis adjusting for explanatory variables showed no differences in the hazard ratio between screened and non-screened groups. Furthermore, it is worth noting that the expected oral cancers and oral cancer mortality among non-attendees were modelled based on previous findings, not calculated directly. Therefore, this study falls short in demonstrating that oral cancer screening saves lives by reducing mortality.

Another study from Taiwan [35] combined three government databases (screening, cancer, and death registries) to obtain real world evidence for the effectiveness of the oral mucosal screening program for mortality reduction as well as early-stage diagnosis. When considering the individual screening diagnosis, the proportion of early stages in patients who were confirmed with cancer at their first screening was similar to cancer patients without any prior screenings (26.3% vs. 27.8%). Hence, there does not appear to be any obvious stage-shift when participants present themselves for cancer screening.

Taken together, these studies do not demonstrate that screening has a clear benefit in reducing mortality from oral cancer.

### 4.3. Cost-Effectiveness of Oral Cancer Screening

Due to differences in habits, healthcare systems, and costs, it is evident that strategies to address oral cancer must consider regional variations in risk factors, incidence rates, population demographics, and healthcare services. There is no one-size-fits-all approach. In an attempt to evaluate the feasibility of implementation of screening programs, particularly in high-risk populations, several cost-effectiveness, cost-utility and modelling analyses have been undertaken [36,37].

According to a Cochrane review [38], the Kerala study is the only RCT that assessed the effectiveness of current screening methods in decreasing oral cancer mortality, and hence can be reliably used as the basis for health economic evaluations. The cost per screening examination was only $6 per person and the incremental cost per life-year saved was US $835 for all individuals and US $156 for those at high risk. Despite the significant findings of the Kerala study, the high risk of bias in the trial has limited the validity and translatability of the findings, calling for further experimental evidence. A more recent study using the Markov modelling approach to estimate the cost and health outcomes of four different approaches, including conventional oral examination, toluidine blue staining, oral cytology, and light-based detection, suggested that the most cost-effective approach in India and low-to-mid income countries would be conventional oral examinations at 10-year intervals for oral screening in high-risk populations above 30 years of age [36].

It is clear that these estimates from low-income and middle-income countries with high oral cancer incidence are not generalizable to western society. One modelling study from the UK [37] examined various screening scenarios within the British health system. Invitation screening and opportunistic screening in either general dental practice or medical practice were never cost-effective options. Despite considerable uncertainty in the parameters used (e.g., malignant transformation rate, disease progression, patterns of self-referral and costs), the study concluded that, overall, high-risk opportunistic screening by a general dental or medical practitioner in primary care settings may be cost-effective, particularly if targeted to younger age groups, particularly 40–60 year olds. A subsequent analysis from the United States suggested that a community-based screening program targeting high-risk males was likely to be cost-effective [39]. The factors commonly included in risk stratification to identify high-risk populations are age, alcohol consumption, and smoking [40].

In summary, while screening is an important public health measure, there is currently insufficient evidence to suggest that screening alone can reduce mortality from oral cancer. Similar conclusions were reached by government bodies across the world and none have found the evidence sufficiently robust to recommend screening as a population health approach [41].

### 4.4. Potential Cost-Effective Alternatives

There are two important characteristics that set oral cancer detection apart from other cancer screenings. Firstly, the process is quick, inexpensive, and requires no special instruments due to the oral cavity’s easy accessibility. Secondly, the mouth is home to the most common disease globally—dental caries—affecting half of the world’s population, as well as to diseases of remarkable clinical significance such as periodontitis. Hence, while oral cancer screening may not be cost-effective per se, it could be integrated with numerous oral health initiatives targeting highly prevalent, albeit generally non-life-threatening, oral conditions. This integration is especially relevant given the newly established link between the oral microbiota, the microenvironment, and cancer development [42]. Thus, combining oral health and cancer screening programs could have a synergistic effect.

Additionally, recent advancements in telehealth and artificial intelligence may facilitate oral cavity inspections in underserved areas where there is a shortage of trained dentists [43]. Teledentistry can provide remote consultations, diagnostics, and even preventive care through digital platforms, thus improving access to oral healthcare. This approach can enhance detection and treatment of oral health issues, potentially reducing the burden of oral diseases and improving overall health outcomes.

Yet, screening is costly, and governments demand strict evidence of benefits and cost-effectiveness before implementing a program [41]. It is also questionable whether early detection through screening (often referred to as secondary prevention) is the best use of public funds for a cancer with well-known risk factors. Oral cavity cancer is largely related to lifestyle, and although it can be easily detected and diagnosed at early stages through a 5-min visual inspection of the oral mucosa, actual figures concerning its primary prevention are dismal. While tobacco control efforts have had remarkable success in western countries (which has coincided with a reduction in oral cancer cases, at least incidentally), the areca nut economy has been rapidly growing in recent decades [44]. Researchers and clinicians have convincingly shown that areca nut alone, without tobacco, is an independent risk factor for oral cancer [45]. It is now time to leverage these data to nudge policymakers to consider appropriate preventive and cessation strategies for areca nut chewing habits, and more broadly, to known modifiable risk factors for oral cancer.

## 5. Role of General Dentists and Technology in Reducing the Burden of Oral Cancer

Nearly half of oral cancer patients experience diagnostic delays, with over 50% presenting at advanced stages of the disease. A meta-analysis indicates that a longer interval from the first symptom to diagnostic referral increases the risk of advanced-stage cancer and mortality [46]. Encouragingly, oral cancer detection can be highly effective through simple visual examinations, suggesting that routine clinical assessments could facilitate early diagnosis and consequently reduce the cancer burden.

### 5.1. The Key Role of General Dentists

Due to the shortage of specialists and the critical role of dentists as frontline dental professionals, the primary responsibility for detecting suspicious lesions falls on general dental practitioners (GDPs). Unfortunately, many GDPs often overlook the assessment and diagnosis of oral mucosal diseases and may not be adequately trained to identify early precursor lesions [47]. Obstacles to routine oral examinations by GDPs include limited knowledge and experience; supporting this view, research indicates that general dentists frequently lack confidence and perform inadequately in addressing suspicious lesions [48].

Crucially, inadequate skills, knowledge, and confidence among GDPs in detecting mouth cancer and precursor lesions may lead to insufficient preventive education for patients, which is essential for primary prevention. As dental practitioners we see our patients regularly and have the opportunity to both initiate discussions about smoking, alcohol, betel quid use, and diet, and provide advice about smoking or chewing cessation, reducing alcohol consumption, and good diet [5].

Enhancing GDPs’ knowledge and awareness of early lesions, as well as improving their ability to conduct thorough examinations and make appropriate referrals, has been shown to reduce diagnostic delays and decrease case fatality rates [49,50]. Therefore, reforming dental and oral health curricula appears crucial in efforts to reduce oral cancer mortality. The global status quo of inadequate knowledge, skills, and awareness among GDPs in oral cancer detection underscores the urgent need for curriculum improvements in dental education [50]. With this in mind, we have recently ignited a change in the classification of premalignant disorders to simplify and streamline the detection of early cancer by emphasizing only the most crucial pathologies—the ones that general dentists need to be trained to recognize effectively [51]. This approach reduces the complexity and breadth of clinical manifestations of precancerous diseases that require immediate action and may prove vital for optimizing the training of GDPs by allowing them to focus on the clinical appearance of oral cancer instead. Typically, early-stage lesions most often appear as red [52], as well as white or mixed red-white lesions [53] (which overlap with the clinical manifestation of premalignant lesions), are often painless (though they may cause discomfort), and progress with features such as ulceration, nodularity, and tissue attachment, which may be painful. Ulceration, characterized by an irregular floor and margins and hardness upon palpation (“induration”), is a typical sign of oral squamous cell carcinoma (OSCC) [53]. The posterior lateral border of the tongue and the floor of the mouth are the most common sites of OSCC, accounting for approximately 50% of all cases, followed by the soft palate, the gingiva, the buccal mucosa, and the hard palate [54]. In general, exophytic (outward-growing) oral cancers are relatively straightforward to detect, and can often be identified early by dentists. In contrast, endophytic (inward-growing) malignancies and those presenting with aspecific features may be more challenging to diagnose, even for specialists, leading to potential delays in detection. In this context, diagnostic aids and risk prediction biomarkers [55] may prove useful in general dental practice. Hence, it is important for oral cancer educators to focus on the actual clinical manifestations of oral malignancies rather than diverging into discussions on OPMDs.

### 5.2. Diagnostic Adjuncts, Telehealth, and Machine Learning for Image Analysis

A range of devices and techniques is now available to help practitioners visualize clinical changes in the oral cavity—overall referred to as diagnostic adjuncts. These methods can be used chairside to assess oral mucosal abnormalities, often providing real-time results at the point of care. They can assist practitioners in making earlier referrals to specialists for further investigation. The value of these adjuncts, however, is unclear. A recent umbrella review concluded that there is limited evidence to recommend the use of chemiluminescence, tissue autofluorescence tools, and vital staining as primary diagnostic methods, though they may serve as adjuncts to conventional oral examination [56]. Cytology techniques and narrow-band imaging can be employed as non-invasive adjunctive tools for detecting OSCC and monitoring the malignant transformation of OPMD. However, their potential role in screening remains uncertain.

Recently, our group conducted the first survey in remote areas of Indonesia to assess oral premalignant conditions and found a high prevalence in West Papua and West Kalimantan [57]. These areas face unique healthcare challenges, including limited access to specialized medical and dental care. Dentists in rural and underdeveloped areas are also particularly disadvantaged, possessing less knowledge and skill in early detection compared to their counterparts in capital cities. This disparity is largely due to the difficulty in accessing professional development centers, which are predominantly located in urban areas. In this context, the implementation of telemedicine and collaborative software emerges as a transformative solution, offering a beacon of hope for enhancing cancer care in these underserved regions. In these areas, virtual training programs on oral cancer early detection are particularly useful [58].

Even though dentists may become better trained at detecting cancer early (with or without diagnostic aids), rural and remote areas would still remain underserved by dental practitioners. In this regard, the use of telehealth for remote consultation and the integration of artificial intelligence in clinical image interpretation could be a game changer. Several platforms already support doctor-to-patient and peer-to-peer consultations via teledentistry, such as Teledentix (https://get.teledentix.com, accessed on 17 August 2024), FirstSightDental^™^ (https://www.stemmresearch.com/aidental, accessed on 17 August 2024), SmileSnap (https://www.smilesnap.com, accessed on 17 August 2024), and this trend has increased with the recent COVID-19 pandemic. More recently, seminal studies have demonstrated the remarkable accuracy of AI-assisted clinical imaging in detecting oral cancer and OPMDs [59]. While this technology is still in its early stages, its potential is vast. Changes in the color and texture of the oral mucosa can be used relatively easily to train machine learning algorithms to recognize oral mucosal pathology, particularly oral cancer and pre-malignant diseases [60]. Examples of AI-powered software currently used chairside in dental practice include Colleague (CoTreat, https://www.cotreat.ai, accessed on 17 August 2024), Second Opinion (Pearl, https://www.hellopearl.com/, accessed on 17 August 2024), and DentrixDetect (VideaHealth, https://www.dentrix.com/, accessed on 17 August 2024), which could leverage their technology for the detection of mucosal disease.

In summary, while it is evident that GDPs bear significant responsibility for opportunistic oral cancer screening, they often feel ill-equipped and perform inadequately when addressing suspicious lesions. As allocating resources for cancer care is crucial in healthcare policy formulation, it would be sensible to begin efforts to reduce oral cancer mortality by reforming oral health curricula and by leveraging emerging technologies to develop reliable chairside tools.

## 6. Conclusions

Screening and early diagnosis have been a cornerstone of preventive medicine for reducing cancer morbidity and mortality. However, the efficacy of these programs, including for oral cancer, is increasingly being questioned. While cancer screening aims to detect cancer in its early stages, the evidence supporting a significant reduction in mortality rates is limited and fraught with methodological challenges. The biases inherent in screening processes, such as lead time bias and length bias, can create an illusion of improved survival without genuinely extending the lifespan of patients. As a consequence, the benefits of screening tests are often overstated.

Specifically for oral cancer screening, despite its potential for opportunistic assessment due to the easy accessibility of the mouth and the frequency of dental visits, there is limited evidence of its effectiveness in reducing mortality at a population level, and research tends to support screening policies only in high-risk groups.

Studies from regions such as India, Taiwan, and Cuba indicate that while early-stage cancer detection may increase, it does not necessarily translate into significant reductions in cancer deaths. This underscores the complexity of determining whether early detection truly “saves lives” as widely publicized. The effectiveness of oral cancer screening programs is further complicated by the challenges of accurately estimating benefits. Methodological biases such as selection bias, false positives, and population heterogeneity affect the perceived efficacy of these programs. Additionally, the cost-effectiveness of such programs varies by region and is influenced by the availability of healthcare resources and compliance rates.

The role of general dentists in oral cancer screening is crucial due to their frequent patient interactions and the simplicity of visual oral examinations. However, many general practitioners lack the necessary training and confidence to effectively identify and refer suspicious lesions, contributing to diagnostic delays and advanced-stage presentations. Improving GDPs’ knowledge and skills is essential for reducing oral cancer mortality. This can be achieved through enhanced dental education curricula, the use of diagnostic adjuncts to assist practitioners in decision-making, and by leveraging artificial intelligence and telehealth in rural and remote areas.

In conclusion, while screening remains a vital public health measure, the evidence supporting its ability to reduce mortality for oral cancer is limited. To improve the effectiveness of cancer screening, particularly for oral cancer, a multifaceted approach that includes reforming dental education, addressing methodological biases, and integrating oral health initiatives is necessary. This integrated approach, supported by advancements in new technologies including telehealth and artificial intelligence, can enhance the early detection and treatment of oral cancers, ultimately improving patient outcomes. Further research is needed to better understand the natural history of the disease and to develop clinical methods for identifying suspicious oral lesions that can progress to malignancy.
